# Supplementary material for: Wheat Leaf Rust Effector Pt48115 Localized in the Chloroplasts and Suppressed Wheat Immunity
Source: J Fungi (Basel). 2025 Jan 20;11(1):80. doi: 10.3390/jof11010080 (PMC11766619; doi:10.3390/jof11010080)
Supplement: Supplementary file 1 [file jof-11-00080-s001.zip › jof-3373400-supplementary.pdf]

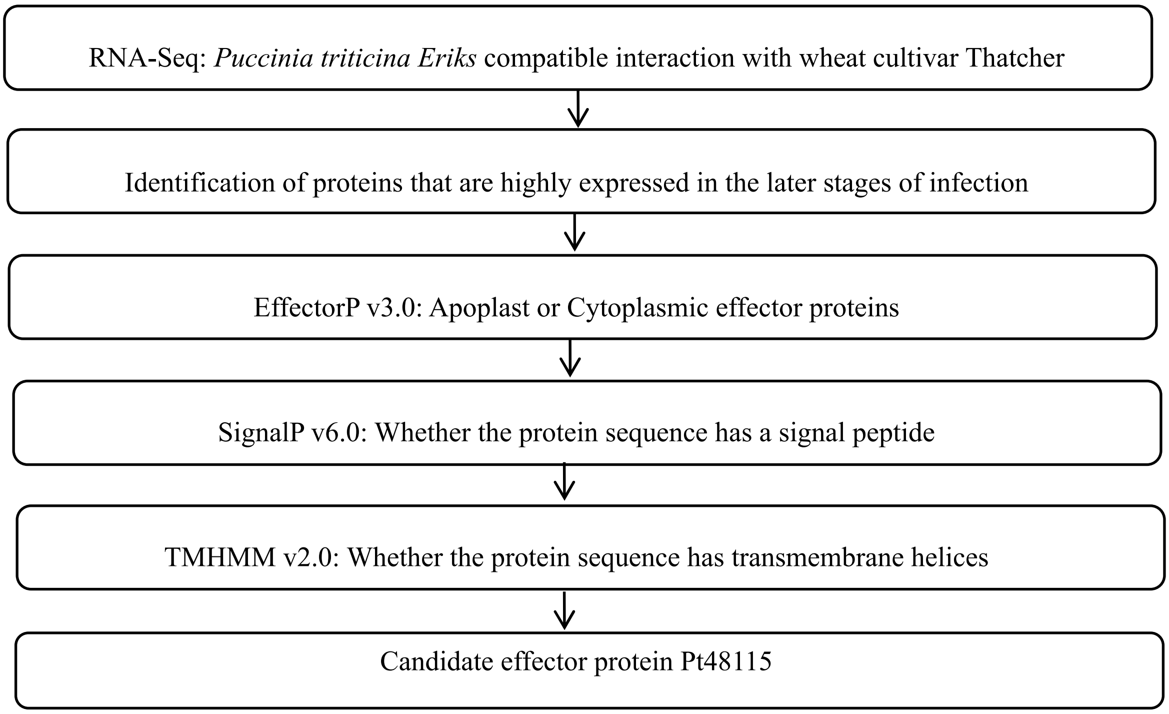

Figure S1. Procedures for screening effector proteins

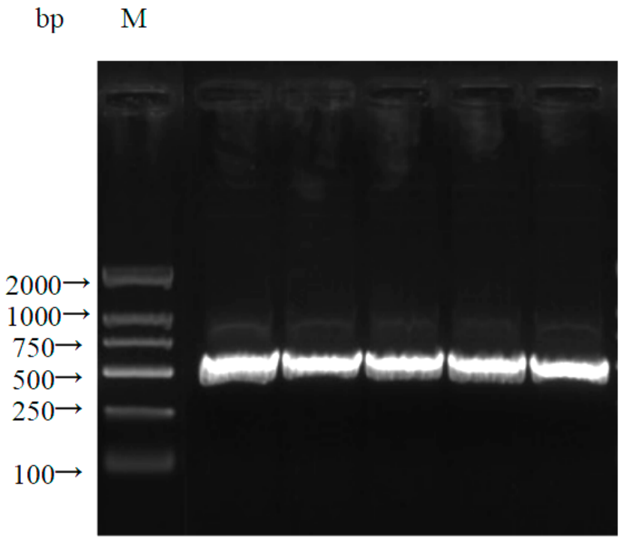

Figure S2. The ORF sequence of Pt48115 was amplified by PCR. M：DNA Marker

```
              10        20        30        40        50        60
1      ATGCGCTCTCGACAACCATCCCACCTCTTCCTGTATTGCCTGTTGGCGCTGGTATCAGATTCA
1       M  R  S  R  Q  P  S  H  L  F  L  Y  C  L  L  A  L  V  S  D  S

              73        83        93        103       113       123
64     GCTCCTCTATCGACTCCTTCAGCACCGCAAACATGCACGTATGTTTATCAGCCTATCACCACG
22      A  P  L  S  T  P  S  A  P  Q  T  C  T  Y  V  Y  Q  P  I  T  T

              136       146       156       166       176       186
127    AGTGGTGATCCAACAGGGAACCAAATGACTTGCCGCAATGCTCAAAGTCCACAAAGGTTCTTC
43      S  G  D  P  T  G  N  Q  M  T  C  R  N  A  Q  S  P  Q  R  F  F

              199       209       219       229       239       249
190    ATCTGTGATCAGAAATCATGTGAAGGCACAAGAAAATGTACGAATTGTGTATCGCAGACTACC
64      I  C  D  Q  K  S  C  E  G  T  R  K  C  T  N  C  V  S  Q  T  T

              262       272       282       292       302       312
253    AATGTCTCCGTGAACTCGATTGAATGCGCAAAATATTACGTAGATGAAAACCAGAAAACAACA
85      N  V  S  V  N  S  I  E  C  A  K  Y  Y  V  D  E  N  Q  K  T  T

              325       335       345       355       365       375
316    AATTGCTGGACATCGGGCGATGAGCAGTTTACCTGCTACGATCAGTGTACCGGGGCCGCTGTC
106     N  C  W  T  S  G  D  E  Q  F  T  C  Y  D  Q  C  T  G  A  A  V

              388       398       408       418       428       438
379    TGCTCGAGTTGCACTCTAGACGAACAAACTCCTTCGATTTCAAACTGTGTCAGCAACTCCTCA
127     C  S  S  C  T  L  D  E  Q  T  P  S  I  S  N  C  V  S  N  S  S

              451       461       471       481       491       501
442    CCAAAACATGGATCCAAAGCCTCACGTGCTTCTAGCGCCAGCACAAGTAACCCAGGTGGCGTA
148     P  K  H  G  S  K  A  S  R  A  S  S  A  S  T  S  N  P  G  G  V

              514       524
505    TCAACGTGGCTTTCCTGGAATTGA
169     S  T  W  L  S  W  N  *
```

Figure S3. Amino acid and protein sequence of Pt48115. Red frame: the sequence of signal peptides; Green frame: the sequence of transit peptides; asterisk (*): The termination codon is not translated into proteins

A B

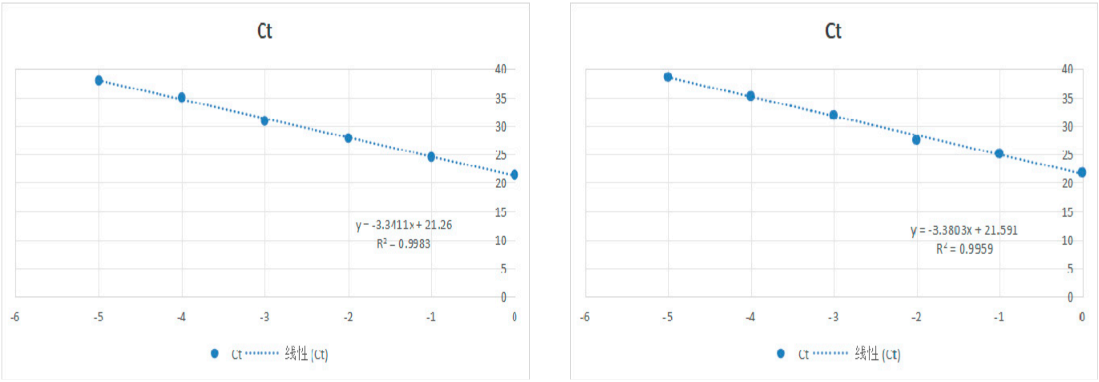

Figure S4. The PCR amplification efficiencies of the primer pairs.

A： *Pt Actin* primer pairs B：*Pt48115* primer pairs

Table S1 Primers used in this study

Primer	Sequence (5'-3')	Application
pMD19-T-Pt48115-F	ATGCGCTCTCGACAACCATC	Cloning of the target gene
pMD19-T-Pt48115-R	TCAATTCCAGGAAAGCCACG	Sequence polymorphism analysis
INFI-Pt48115-F	GTCAGCACCAGCTAGCATCGATATGCGCT CTCGACAACCATC	Verification of inhibition of PCD of *Nicotiana benthamiana*
INFI-Pt48115-R	CGCCCTTGCTCACCATCCCGGGATTCCAG GAAAGCCACG	
qPCR-Pt48115-F	CATCCCACCTCTTCCTGTATTG	Detection of Pt48115 expression
qPCR-Pt48115-R	TCTTGTGCCTTCACATGATTTCT	
pEDV6-Pt48115-F	CACCATGCGCTCTCGACAACCATC	Determination of callose deposition and H_2O_2 accumulation
pEDV6-Pt48115-R	TCAATTCCAGGAAAGCCACG	
SP-Pt48115-F	CCGGAATTCATGCGCTCTCGACAACCATC	Validation of signal peptide secretion function
SP-Pt48115-R	CCGCTCGAGTGAATCTGATACCAGCGCCA	
ΔCTP1-Pt48115-F	CATTTACGAACGATACTCGAGATGCAGAC TACCAATGTCTCCGT	Subcellular localization analysis with removal of transport peptides
ΔCTP1-Pt48115-R	CACCATCACTAGTACGTCGACATTCCAGG AAAGCCACGTTG	
Pt48115-F1	GTCAGCACCAGCTAGCATCGATGCTCCTC TATCGACTCCTTCAG	Sequence of major toxic domains
Pt48115-R1	CGCCCTTGCTCACCATCCCGGGTCAATTC CAGGAAAGCCACG	
Pt48115-R2	CGCCCTTGCTCACCATCCCGGGGGAGTTG CTGACACAGTTTGAA	
Pt48115-R3	CGCCCTTGCTCACCATCCCGGGGGTAAAC TGCTCATCGCCC	
Pt48115-F2	GTCAGCACCAGCTAGCATCGATGCTCCTC TATCGACTCCTTC	
Pt48115-R4	CGCCCTTGCTCACCATCCCGGGGACATTG GTAGTCTGCGATA	
